# *PCK1* Deficiency Shortens the Replicative Lifespan of *Saccharomyces cerevisiae* through Upregulation of *PFK1*

**DOI:** 10.1155/2020/3858465

**Published:** 2020-02-12

**Authors:** Yuan Yuan, Jia-ying Lin, Hong-jing Cui, Wei Zhao, Hui-ling Zheng, Zhi-wen Jiang, Xing-dong Xiong, Shun Xu, Xin-guang Liu

**Affiliations:** ^1^Guangdong Provincial Key Laboratory of Medical Molecular Diagnostics, Institute of Aging Research, Guangdong Medical University, Dongguan, China; ^2^The Scientific Research Center of Dongguan, Guangdong Medical University, Dongguan, China; ^3^Institute of Biochemistry & Molecular Biology, Guangdong Medical University, Zhanjiang, China

## Abstract

The cytosolic isozyme of phosphoenolpyruvate carboxykinase (*PCK1*) was the first rate-limiting enzyme in the gluconeogenesis pathway, which exerted a critical role in maintaining the blood glucose levels. *PCK1* has been established to be involved in various physiological and pathological processes, including glucose metabolism, lipid metabolism, diabetes, and tumorigenesis. Nonetheless, the association of *PCK1* with aging process and the detailed underlying mechanisms of *PCK1* on aging are still far to be elucidated. Hence, we herein constructed the *PCK1*-deficient (*pck1*Δ) and *PCK1* overexpression (*PCK1 OE*) *Saccharomyces cerevisiae*. The results unveiled that *PCK1* deficiency significantly shortened the replicative lifespan (RLS) in the *S. cerevisiae*, while overexpression of *PCK1* prolonged the RLS. Additionally, we noted that the ROS level was significantly enhanced in *PCK1*-deficient strain and decreased in *PCK1 OE* strain. Then, a high throughput analysis by deep sequencing was performed in the *pck1*Δ and wild-type strains, in an attempt to shed light on the effect of *PCK1* on the lifespan of aging process. The data showed that the most downregulated mRNAs were enriched in the regulatory pathways of glucose metabolism. Fascinatingly, among the differentially expressed mRNAs, *PFK1* was one of the most upregulated genes, which was involved in the glycolysis process and ROS generation. Thus, we further constructed the *pfk1*Δ*pck1*Δ strain by deletion of *PFK1* in the *PCK1*-deficient strain. The results unraveled that *pfk1*Δ*pck1*Δ strain significantly suppressed the ROS level and restored the RLS of *pck1*Δ strain. Taken together, our data suggested that *PCK1* deficiency enhanced the ROS level and shortened the RLS of *S. cerevisiae* via *PFK1*.

## 1. Introduction

Aging is characterized by a progressive loss of physiological integrity, which was driven by a variety of contributing factors, including DNA damage, epigenetic shifts, and altered RNA and protein profiles [1–3]. The deterioration inevitably impairs tissue function and increases susceptibility to disease and death and has been demonstrated to be the primary risk factor for major human pathologies, including cancer, diabetes, cardiovascular disorders, and neurodegenerative diseases [4]. Current evidences have demonstrated that alterations in energy metabolism were closely linked to the aging process and aging-associated diseases [5]. Nevertheless, the association of the energetic metabolism with aging and the underlying molecular mechanism were remain elusive [6].

*S. cerevisiae* is a single-celled organism with a short lifespan, which has been an ideal model organism for aging research [7]. In addition, because of the genetic and biochemical capacities and its role as a workhorse in food production and biotechnology, budding yeast has been a major eukaryotic model for the study of metabolic network structure and function as well [8]. Moreover, the rate of metabolism is a factor in determining the longevity of *S. cerevisiae* [9]. Therefore, we herein probed into the effect and underlying mechanism of *PCK1* on the aging process in *S. cerevisiae*.

*PCK1* is a key enzyme in the gluconeogenesis process, which catalyzes the generation of phosphoenolacetone from oxaloacetate acid. *PCK1* is widely expressed in a series of tissues, including liver, kidney, and white and brown adipose tissues [10]. *PCK1* has been demonstrated to be a multifunctional gene and was closely related to gluconeogenesis, obesity, and diabetes [11, 12]. Recent studies have shown that *PCK1* expression is significantly reduced in aging nematodes after the peak reproductive period, and *PCK1* expression is downregulated in skeletal muscle and liver in aging mammals [13–16]. Nonetheless, the effect and detailed molecular mechanism of *PCK1* on aging process are still far to be elucidated.

Recently, the relationship between metabolism and aging has gained increasing attention. The aging process is accompanied by a series of changes in the expression levels of metabolism-related genes. In this study, we identified differentially expressed genes between the *PCK*1-deficient strain and the wild-type strain utilizing high throughput analysis, and we sought to shed light on the roles of the effects of *PCK1* on aging process and illuminate the underlying molecular mechanisms.

## 2. Materials and Methods

### 2.1. Yeast Strains and Culture

The yeast strains used in this study are listed in Table 1. All strains were isogenic to BY4742 and were stored in liquid yeast peptone dextrose (YPD; 1% yeast extract, 2% peptone, and 2% glucose; Oxoid, Basingstoke, UK) medium mixed with equal volume of 50% (v/v) glycerol at −80°C. For all experiments, cells were removed from frozen stock and streaked onto solid YPD plate incubation overnight at 30°C. Then, the single colonies were picked and grown in liquid YPD until the exponential phase using an orbital shaker where yeast cells were shaken at 30°C and 180 rpm [17].

### 2.2. *PCK1* Deletion and Overexpression Strains Construction

Wild-type yeast strains (BY4742) were a gift from Dr. Matt Kaeberlein (University of Washington, USA). The strains used in this study were derived from BY4742 (MATα *his3*Δ*1 leu2*Δ0 *lys2*Δ0 *ura3*Δ0). The *PCK1*-deficient strain (BY4742 *pck1*:URA3) was produced through PCR-mediated one-step gene disruption (primers: 5′-AAACTCACG CAACTAATTAT TCCATAATAAAATAACAACAGATTGTACTGAGAGTGCAC-3′ and 5′-TTTTTTTTGGATTGAACATATCGAAGGAACATGTTTCGTCTGTGCGGTATTTCACACCG-3′) using the plasmid pRS306 with *URA3* as the selectable marker [18]. The *pck1*Δ mutants were constructed as previously described. Briefly, *URA3* cassette was amplified by polymerase chain reaction (PCR) from pRS306 vector using the following primers: 5′-TGTTTTAACTGGGA-AAAAGCGGAACAATTGGGCCT.

TACAAGATTGTACTGAGAGTGCAC-3′ (forward) and 5′-CGTGCTCATCAATGT.

GAACAAATTATTAAATACAAGCGTCTGTGCGGTA-TTTCACACCG-3′ (reverse) (Invitrogen, USA). Then, the PCR products were transformed into BY4742 to replace *PCK1* by homologous recombination, and the transformants were selected on SD URA medium (Clontech, Mountain View, CA, USA). The *pck1*Δ cells were verified by PCR using the following primers: 5′-TGTGGCTGTCGTTTCGTGG-3′ (forward) and 5′-TACAGTTTCCACTGCGAACACA-3′ (reverse) (Invitrogen, USA).

The *PCK1* overexpression plasmid (pRS306-*PCK1-OE*) was constructed by inserting a 1900 bp *Bam*HI-*Eco*RI fragment and a 1417 bp *Eco*RI-*Cla*I fragment amplified from yeast genomic DNA into the *Bam*HI and *Cla*I sites of pRS306. In addition to the ORF of *PCK1*, ∼533 nucleotides of upstream sequence and ∼300 nucleotides of downstream sequence were amplified [19, 20]. Thus, expression of *PCK1* would be driven by its natural promoter. The overexpressing *PCK1* strain was constructed by transforming wild-type yeast cells with *Hpa*I-digested plasmid pRS306-*PCK1-OE*.

*PCK1* and *PFK1* double gene disruptions (*pck1*Δ*pfk1*Δ) were constructed by mating single gene deleted yeast strains containing the different selectable markers (BY4742 *pck1*:*URA3* and BY474*1 pfk1*:*LEU2*), and further individual meiotic tetrads were dissected under an optical microscope and grown on yeast YPD plates at 30°C.

### 2.3. RNA Isolation, cDNA Synthesis, and Real-Time PCR

Total RNA was isolated from yeast cells using a Yeast RNAiso Kit (Takara, Otsu, Shiga, Japan) followed by cDNA synthesis using a Transcriptor First-Strand cDNA Synthesis Kit with gDNA Eraser (Takara, Otsu, Shiga, Japan). Real-time PCR was performed using SYBR PremixEx Taq (Takara, Otsu, Shiga, Japan). Data were normalized to the internal control *PRP*8 [20, 21].

### 2.4. Growth Determination

The growth was assessed in the cell culture plates using a Bioscreen CMB instrument (Finland) [19, 22, 23]. Yeast cultures were prepared from a single colony and diluted in 10-fold series. The working volume in the wells of the Bioscreen plate was 300 *μ*L with or without glucose, comprising 100 *μ*L of culture medium (optical density (OD) 600 of approximately 0.04) and 200 *μ*L of liquid YPD medium. The OD of the cell suspensions was measured automatically at 600 nm at regular 2-hour intervals at 30°C for 2 days. The data were calculated as the average absorbance of three duplicates for each type of culture medium and used to construct growth curves for each strain studied by plotting at 32-hour intervals. The results were subjected to the Friedman test (SPSS 12.0) with a significance level of 5%.

### 2.5. Spot Assay

Yeast cells were cultured to exponential phase (OD600 = 2.0) in liquid YPD followed by a 10-fold dilution with sterile water. Four additional 10-fold serial dilutions were performed, and 3.5 *μ*L of each dilution was inoculated onto solid YPD plates supplemented with or without glucose. Templates were incubated at 30°C for 2∼5 days for colony formation [17].

### 2.6. Glucose, Pyruvic Acid, Citrate, and Lactate Level Assay

Yeast cells in exponential phase were harvested and washed twice with cold sterile water followed by resuspension in lysis buffer containing acid-washed glass beads and 20 cycles of 10 seconds of vortexing plus 20 seconds of cooling. After centrifugation at 12000 rpm for 15 minutes, the supernatants were collected and used for glucose and pyruvic acid, citrate, and lactate level assay. The protein concentration was determined using the BCA protein assay kit (Sangon Biotech, Shanghai, China) following the manufacturer's instruction. The intracellular glucose and pyruvic acid level were determined using a glucose assay kit (Abcam, Shanghai, US) and pyruvic acid, citrate, and lactate level assay kit (Jiancheng, Nanjing, China).

### 2.7. Replicative Lifespan (RLS) Assay

RLS assay was performed as previously described [24]. Briefly, cells were thawed from frozen stocks and grown on a fresh YPD plate overnight and then patched onto the second fresh YPD plate and incubated for about 24 hours. Cells were then restruck on the third fresh YPD plate and incubated for about 12 hours, and then the virgin buds were isolated and used for RLS analysis [17].

### 2.8. Analysis of ROS Production

The generation of intracellular ROS in yeast cells was detected using the oxidant-sensitive probe 2′,7′-dichlorodihydrofluorescein diacetate (DCFH-DA) [25]. Exponential phase cells were incubated for 12 hours in liquid medium (YPD). Cells were then harvested and incubated with 5 mM DCFH-DA in YPD medium for 1 hour at 30°C. Cells were washed and subjected to flow cytometric analysis. The excitation wavelength was 488 nm and the observation wavelength was 525 nm for green fluorescence [26].

### 2.9. Statistical Analysis

All experiments were repeated at least three times. Data were expressed as the mean ± standard deviation (SD). Statistical significance was assessed by calculating *p* values using a two-tailed Student's *t*-test or Wilcoxon rank sum test. A value of *p* < 0.05 was considered statistically significant.

## 3. Results

### 3.1. *PCK1* Deletion Shortened the Lifespan of *S. cerevisiae*

**Figure 1:**
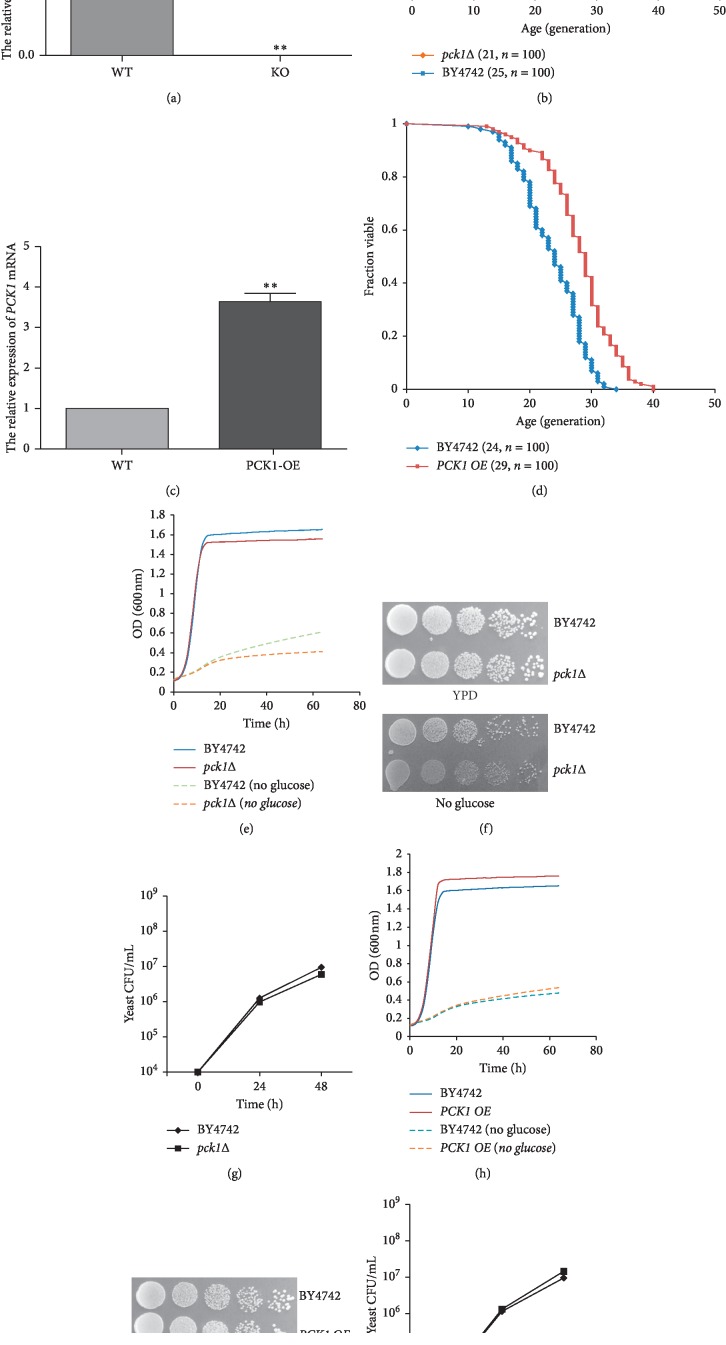
*PCK1* deletion shortened the lifespan of *S. cerevisiae*. (a) Real-time qPCR was used to detect the *PCK1* expression levels in BY4742 and *pck1*Δ strain. Columns, mean of at least three independent experiments; bars, SEM. ^*∗∗*^*P* < 0.01, comparison between two groups as indicated. (b) RLS curves of BY4742 and *pck1*Δ strains. The survival curves were generated from lifespan data, and BY4742 was considered as the control. Numbers in parentheses are the median RLS and cell number. (c) Real-time qPCR was utilized to monitor the *PCK1* expression levels in BY4742 and *PCK1 OE* strain. Columns, mean of at least three independent experiments; bars, SEM. ^*∗∗*^*P* < 0.01, comparison between two groups as indicated. (d) RLS curves of BY4742 and *PCK1 OE* strains. The survival curves were generated from lifespan data, and BY4742 was considered as the control. Numbers in parentheses are the median RLS and cell number. (e) The growth curves of BY4742 and *pck1*Δ yeast cells based on monitoring (A600) at indicated times with or without glucose. (f) BY4742 and *pck1*Δ yeast cells were diluted (1 : 10) sequentially and cultured on YPD plates with or without glucose. (g) BY4742 and *pck1*Δ yeast cells were cultured in YPD medium, in all cases, yeast was inoculated at ∼10^4^ CFU/mL. (h) The growth curves of BY4742 and *PCK1 OE* yeast cells based on monitoring (A600) at indicated times with or without glucose. (i) BY4742 and *PCK1 OE* yeast cells were diluted (1 : 10) sequentially and cultured on YPD plates with or without glucose. (j) BY4742 and *PCK1 OE* yeast cells were cultured in YPD medium; in all cases, yeast was inoculated at ∼10^4^ CFU/mL.

To verify the role of *PCK1* in aging process, a *PCK1* deletion (*pck1*Δ) strain was constructed by homologous recombination; the disruption of *PCK1* was confirmed by qPCR (Figure 1(a)). Subsequently, the RLS of the *pck1*Δ strain was determined under an optical microscope. Expectedly, *PCK1* deficiency shortened the RLS of yeast cells by approximately 16% (Figure 1(b)), compared with that of the wild-type strain (BY4742). We further constructed the *PCK1* overexpression strain (*PCK1 OE*), and the overexpression of *PCK1* was confirmed (Figure 1(c)). Consistently, the ectopic expression of *PCK1* significantly extended the RLS of yeast cells by approximately 21%, compared with that of the BY4742 strain (Figure 1(d)).

Additionally, the growth behavior of the *pck1*Δ yeast cells was investigated using the Bioscreen CMB system and the spot assay. The results showed that the growth of the *pck1*Δ strain was significantly inhibited in YPD deprived of glucose (Figures 1(e)–1(g)), compared with that of the BY4742 strain, while overexpression of *PCK1* only conferred a modest effect on the growth of *S. cerevisiae* (Figures 1(h)–1(j)). Taken together, our data further showed that *PCK1* deficiency evidently shortened the lifespan of *S. cerevisiae*.

### 3.2. *PCK1*-Deficient *S. cerevisiae* Enhanced the ROS Levels through Impacting Glucose Metabolism

**Figure 2:**
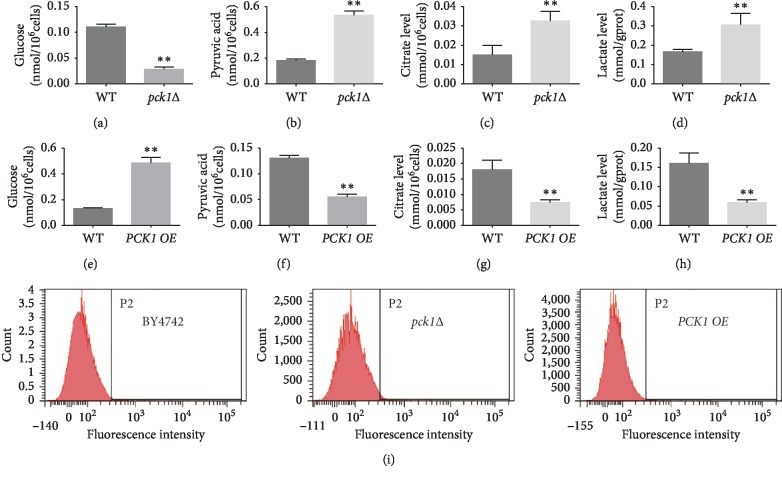
*PCK1*-deficient *S. cerevisiae* enhanced the ROS levels through impacting glucose metabolism. (a)–(d) The production of glucose, pyruvic acid, citrate, and lactate level were examined in BY4742 and *pck1*Δ strain. (e)∼(h) The production of glucose, pyruvic acid, citrate, and lactate level was examined in BY4742 and *PCK1 OE* strain. Columns, mean of at least three independent experiments; bars, SEM. ^*∗*^*P* < 0.05, ^*∗∗*^*P* < 0.01, comparison between two groups as indicated. (i) The ROS level of *PCK1*-deficient *S. cerevisiae*.

*PCK1* was one of the most important enzymes in gluconeogenesis. Thus, the effect of *PCK1* deficiency on glucose metabolism was further verified by evaluation of the yeast cell glucose and pyruvic acid, citrate, and lactate by glucose assay kit and pyruvic acid, citrate, and lactate level assay kits. The results showed that deletion of *PCK1* significantly diminished the glucose level and enhanced the production of pyruvic acid, citrate, and lactate (Figures 2(a)–2(d)). On the contrary, ectopic expression of *PCK1* endowed *S. cerevisiae* with increased glucose level and decreased pyruvic acid, citrate, and lactate (Figures 2(e)–2(h)).

Previous evidence has established that ROS level was closely associated with the cellular senescence, aging, and age-related diseases [27]. In addition, ROS are generated from the reaction during oxygen metabolism. Thus, it is reasonable to speculate that *PCK1* deficiency may cause the abnormal ROS level. We further dug into the effect of *PCK1* on ROS levels in *S. cerevisiae* by flow cytometric analysis. The results unveiled that *PCK1* deficiency significantly enhanced the ROS level, while overexpression of *PCK1* reduced the ROS level (Figure 2(i)), indicating that *PCK1* deficiency might elevate the ROS level through impacting glucose metabolism and thus lead to the shortened lifespan of *S. cerevisiae*.

### 3.3. Identification of Differentially Expressed mRNAs in *PCK1* Deletion Strains

**Figure 3:**
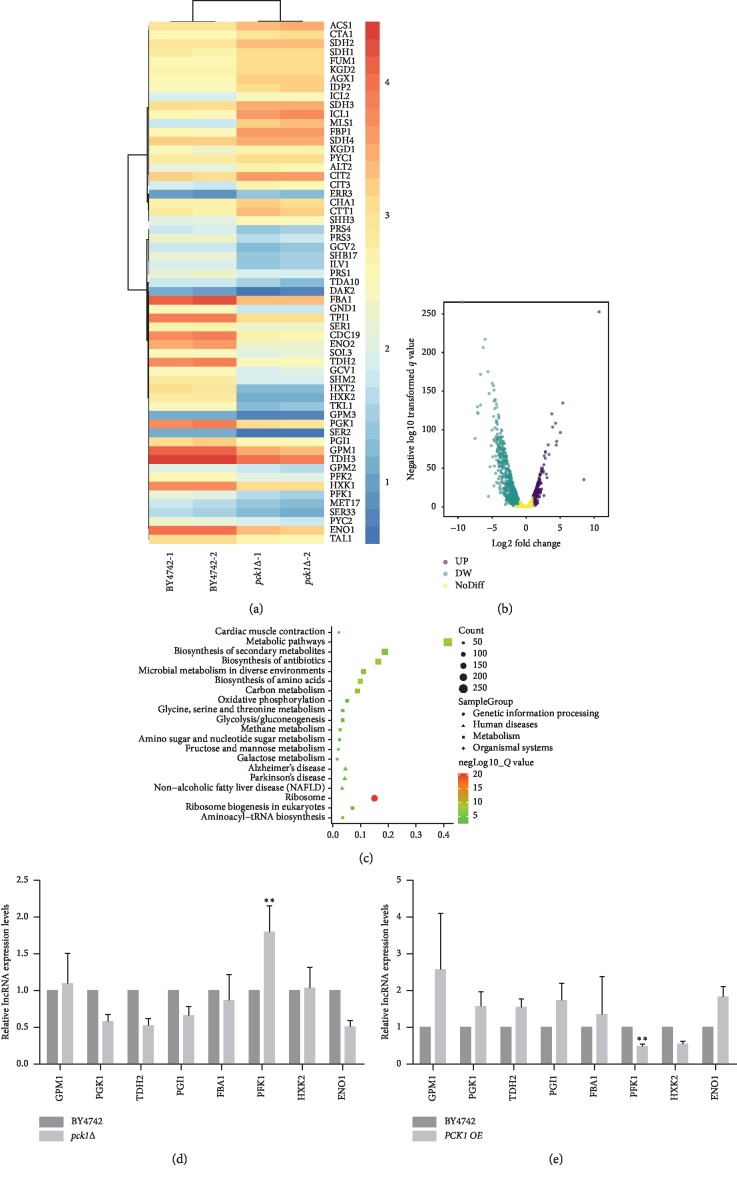
Identification of differentially expressed mRNAs in *PCK1* deletion strains. (a) mRNA expression profiles of BY4742 and *pck1*Δ strain (*n* = 2) were evaluated by deep sequencing. The heat map was generated from the hierarchical cluster analysis to show a distinguishable mRNA expression profile between BY4742 and *pck1*Δ strain. The color is determined by the ratio between the mRNA signal value of BY4742 and *pck1*Δ strain. (b) Volcano map of differentially expressed genes. Each dot in the figure represents a specific gene, with the purple dots indicating significantly upregulated genes, the green dots indicating significantly downregulated genes, and the black dots indicating nonsignificant genes. (c) Differentially expressed gene KEGG pathway enrichment scatter plot. Each dot in the figure is a KEGG pathway, and the ordinate text indicates the pathway name of KEGG, and the classification description is as shown in the right class legend. The abscissa is expressed as the enrichment rate, and the formula is as follows: Enrich_factor = GeneRatio/BgRatio; the color indicates the significance of the enrichment. (d) The mRNA expression levels of *GPM1*, *PGK1*, *TDH2*, *PGI1*, *FBA1*, *ENO1*, *PFK1*, and *HXK2* were examined in BY4742 and *pck1*Δ strains. (e) The mRNA expression levels of *GPM1*, *PGK1*, *TDH2*, *PGI1*, *FBA1*, *ENO1*, *PFK1*, and *HXK2* were examined in BY4742 and *PCK1 OE* strains. Columns, mean of at least three independent experiments; bars, SEM. ^*∗*^*P* < 0.05, ^*∗∗*^*P* < 0.01, comparison between two groups as indicated.

To explore the potential molecular mechanisms of the effect of *PCK1* deficiency on the lifespan of *S. cerevisiae*, we further performed deep sequencing from *pck1*Δ strain and BY4742 group to monitor the mRNA expression profiling. The data revealed 1240 differentially expressed mRNAs between *pck1*Δ strain and BY4742 with a fold change ≥2.0 (*p* < 0.05) (Figures 3(a) and 3(b)). Then, a gene KEGG pathway analysis was performed to evaluate the association among these differentially expressed mRNAs. The results unraveled that the most downregulated mRNAs were enriched in the gluconeogenesis pathways, including *ENO1*, *PGK1*, *TDH2*, and *PGI1*, whereas the upregulated mRNAs were enriched in the glycolysis pathways, including *HXK2* and *PFK1* (Figure 3(c)), indicating the critical role of the glucose metabolism in aging process.

The downregulation and upregulation of partially differentially expressed mRNAs were further confirmed by real-time qPCR in *pck1*Δ strain, *PCK1 OE* strain, and BY4742 strain (Figures 3(d) and 3(e)). The results unraveled that, among the differentially expressed genes, the glycolysis pathways related gene *PFK1* was significantly upregulated in *pck1*Δ strain and downregulated in *PCK1 OE* strain (Figures 3(d) and 3(e)). It is reasonable to speculate that *PFK1* might probably exert a crucial effect on *PCK1*-mediated function in the regulation of *S. cerevisiae* lifespan.

### 3.4. *PFK1* Deletion Restored *PCK1*-Deficient Strains' RLS and Inhibited ROS Level

**Figure 4:**
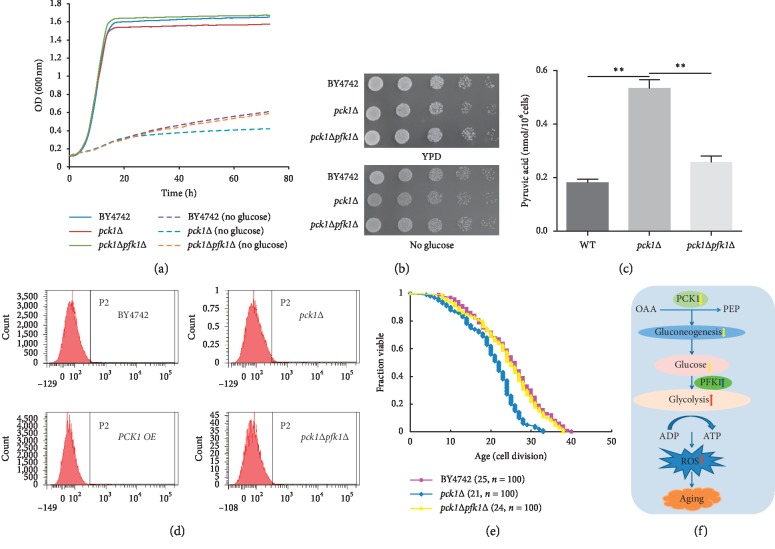
*PFK1* deletion restored *PCK1* deficiency strains RLS by inhibited ROS level. (a) The growth curves of BY474*2*, *pck1*Δ, and *pck1 pfk1*Δ yeast cells based on monitoring (A600) at various time points. (b) BY4742, *pck1*Δ, and *pck1*Δ*pfk1*Δ yeast cells were diluted (1 : 10) sequentially and cultured on YPD plates. (c) The production of pyruvic acid levels was examined in BY4742, *pck1*Δ, and *pck1*Δ*pfk1*Δ strains. Columns, mean of at least three independent experiments; bars, SEM. ^*∗*^*P* < 0.05, ^*∗∗*^*P* < 0.01, comparison between two groups as indicated. (d) BY4742, *pck1*Δ, *PCK1 OE*, and *pck1*Δ*pfk1*Δ yeast cells were cultured for 12 hours and stained with DCFH-DA. Resuspended cells were subjected to flow cytometric analysis. (e) RLS curves of BY4742, *pck1*Δ, and *pck1*Δ*pfk1*Δ strains. Mortality curves were generated from lifespan data, and BY4742 was considered the control. Numbers in parentheses are the median RLS and cell number. (f) Mechanism diagram of *PCK1*-deficient shortened yeast replication lifespan.

Previous literature has demonstrated that *PFK1* exerted a critical role during glycolysis process, and *PFK1* deletion reduced downstream glycolytic intermediates and glucose consumption [28–30]. To probe into the effect of *PFK1* in *PCK1*-mediated function in the regulation of *S. cerevisiae* lifespan, we further constructed the *pfk1*Δ*pck1*Δ strain by deletion of *PFK1* in *PCK1*-deficient strain. Subsequently, the growth, pyruvic acid production, ROS level, and RLS of *pfk1*Δ*pck1*Δ strain were evaluated. The results showed that deletion of *PFK1* in *PCK1*-deficient strain significantly restored the growth in YPD deprived of glucose (Figures 4(a) and 4(b)) and reduced pyruvic acid (Figure 4(c)). Moreover, deletion of *PFK1* in *PCK1*-deficient strain significantly inhibited the ROS level (Figure 4(d)) and restored the RLS of *pck1*Δ strain (Figure 4(e)). Taken together, our data suggested that *PCK1* deficiency enhanced the ROS level and shortened the RLS of *S. cerevisiae* via *PFK1* (Figure 4(f)).

## 4. Discussion

Accumulating lines of evidence have uncovered that glucose metabolism was closely associated with aging process [31–33]. *PCK1* was the first rate-limiting enzyme in the gluconeogenesis pathway and was probably involved in the aging process in *C. elegans*. We herein revealed that *PCK1* deficiency endowed the *S. cerevisiae* with growth inhibition and shortened replicative lifespan, which further verified the close relationship between aging and glucose metabolism.

*PCK1* is a multifunctional gene that is closely related to gluconeogenesis, obesity, and diabetes. Previous studies have reported that *PCK1* was evidently downregulated in skeletal muscle and liver of aged mammals, and overexpression of *PCK1* significantly prolonged the lifespan of nematodes and mice [13–16, 34]. Our demonstration that *PCK1* deficiency dramatically shortened the lifespan of *S. cerevisiae* further verified the crucial effect of *PCK1* in aging process, which was consistent with the above published literature. Replicative lifespan and chronological lifespan are both important issues in aging research. Our data showed that the overexpression of *PCK1* significantly extended the RLS of yeast cells. It has also been reported that the *PCK1* activity extends chronological lifespan [35]. Interestingly, however, the trade-off between reproduction and lifespan, particularly the fact that the mutations that extend lifespan decrease reproduction, has also been reported [36]. In another study with *Daphnia pulex*, a model organism for aging, the effects of fluctuating temperature and food availability on reproduction and lifespan have also been studied and it has been reported that the individuals at the highest food levels usually had the highest reproductive output along with the longest lifespans [37]. In a more recent study, a *S. cerevisiae* mutant with increased chronological lifespan was obtained by evolutionary engineering or adaptive laboratory evolution, and the comparative transcriptomic analysis of the mutant revealed that the mutant had an upregulated respirative-oxidative metabolism [38]. Considering the upregulation of glycolysis-related genes and downregulation of the gluconeogenic genes in the *pck1* deletion mutant in our study, these findings indicate the importance of metabolic changes in the extension of chronological and replicative lifespan in aging studies. Additionally, the link between chronological and replicative lifespan has yet to be clarified at molecular and metabolic levels.

*PFK1* is a key regulatory enzyme of glycolysis. It catalyzes the formation of fructose 1,6-bisphosphate from fructose 6-phosphate (F6P) and adenosine triphosphate (ATP). *PFK1* has been considered as a potential regulator of skeletal muscle insulin sensitivity and altered insulin-stimulated glucose metabolism, and differential expression of phosphofructokinase-1 isoforms also correlates with the glycolytic efficiency of breast cancer cells [39–41]. Nonetheless, the effect of *PFK1* on aging process was largely unknown. Our demonstration that *PFK1* deletion significantly restored the RLS and growth of *PCK1*-deficient strain further enlarged the knowledge of *PFK1* and confirmed the relationship between glycolysis and aging process.

In aggregate, we herein presented lines of evidence that *PCK1* deficiency significantly shortened the RLS and growth in *S. cerevisiae* and further identified *PFK1* as the downstream gene of *PCK1*, which evidently restored the effect of *PCK1* on the RLS of *S. cerevisiae*. Hence, we proposed a hypothesis that the *PCK1* deficiency shortened replicative lifespan of *S. cerevisiae* through *PFK1*, which enhanced the pyruvic acid production and ROS level, finally leading to cellular senescence. Nonetheless, further investigations were still required to fully elucidate the detailed mechanisms underneath the effect of *PCK1* in addition to the *PFK1* pathway.

## 5. Conclusions

In conclusion, our study demonstrates for the first time that *PCK1* deficiency shortened the replicative lifespan of *S. cerevisiae*. *PFK1* is one of the most upregulated mRNA in *pck1*Δ strain. Deletion of *PFK1* in *PCK1*-deficient strain evidently restored the RLS of *pck1*Δ strain.

## Figures and Tables

**Table 1:** Yeast strains used in this study.

Strain	Genotype	Source
BY4742	MATa *his3*Δ*1 leu2*Δ0 *met1*5Δ0 *ura3*Δ0	Gift from Dr.
		Matt Kaeberlein
*pck1*Δ	BY4742 *pck1*:*URA3*	This study
*PCK1 OE*	BY4742 *PCK1 OE*:*URA3*	This study
*pck1*Δ*pfk1*Δ	BY4742 *pfk1:URA3 pck1*:*LEU2*	This study

## Data Availability

All data included in this study are available upon request through contacting the corresponding author.
